# Editorial: Multi-omics research as biomedical interfaces with the engineering and informatics for healthcare

**DOI:** 10.3389/fbioe.2023.1281462

**Published:** 2023-09-04

**Authors:** Yuning Wang, Xiaodan Huang, Kun Qian

**Affiliations:** ^1^ State Key Laboratory for Oncogenes and Related Genes, School of Biomedical Engineering and Institute of Medical Robotics, Shanghai Jiao Tong University, Shanghai, China; ^2^ Australian Institute for Bioengineering and Nanotechnology, The University of Queensland, Brisbane, QLD, Australia

**Keywords:** multi-omics, bioinformatics, diagnostics, sensors, devices, nano-omics

In the era of personalized medicine, precise and timely diagnosis of chronic and serious diseases is crucial to enhance survival rates, which is beneficial for human healthcare. However, most of the current technologies have limitations in clinical diagnostics and management. With the development of multi-omics analysis, transcriptomics, proteomics, and metabolomics have gradually been used to discover important biological molecules and explore their potential correlations with diseases. Therefore, this Research Topic aims to present typical findings in advanced technologies for disease management. In this Research Topic, we have collected five articles, including three articles on proteomics or metabolomics analysis for disease diagnosis and two articles on novel innovations for treatment.

For multi-omics analysis for disease diagnosis, metabolomics directly reflects the physiological and pathological processes. Fan et al. conducted a metabolomics platform to identify diagnostic biomarkers for the early stage of acute aortic dissection with acute lung injury, aiming to address the perturbed pathways underlying disease progression and pathophysiological mechanisms. Ding et al. developed nano-material-based chips for high-performance metabolic analysis by direct metabolic fingerprinting of urine towards kidney stone diagnostics. Proteomic analysis as a promising strategy has been used to obtain a global view of complex issues. Lu et al. launched a temporal quantitative proteomics approach combined with real-time live-cell imaging to discover the molecular changes in virus-host interactions at different hours post-infection. The systematic proteomic analysis provided potential therapeutic biomarkers and biomedical applications for viral treatments.

For novel innovations for disease management, Yang et al. conducted a randomized controlled study to explore the safety and efficacy of remote ischemic conditioning in adult patients with Moyamoya disease who were undergoing revascularization therapy. The study provided a potential treatment method for Moyamoya disease. In another research, Wang et al. developed a method for rapid and accurate human leukocyte antigen typing technology and a supporting algorithm based on the high-resolution mass analysis function of MS and some short fragment PCR amplification target products as typing tags with precise prime. This method addressed the problem of ambiguity in the nucleotide sequence signal overlay generated by the gold standard using polymerase chain reaction sequence-based typing, with advantages in terms of cost and efficiency over existing methods.

In short, the Research Topic shows several representative types of research on advanced strategies for disease diagnosis and management. With the continuous development of technology and difficult issues in urgent need of being solved, more research will promote the progress of this field in the future. We gratefully appreciate the support from the whole Frontiers editorial team and the reviewers. We would like to thank all authors who made great contributions to this Research Topic.

